# Hsa‐miR‐134‐5p predicts cardiovascular risk in circulating mononuclear cells and improves angiogenic action of senescent endothelial progenitor cells

**DOI:** 10.1111/jcmm.18523

**Published:** 2024-07-03

**Authors:** Ting‐Yi Tien, Yih‐Jer Wu, Chiung‐Yin Chang, Chung‐Lieh Hung, Yi‐Nan Lee, Hsin‐I Lee, Yen‐Hung Chou, Chao‐Feng Lin, Chun‐Wei Lee, Cheng‐Huang Su, Hung‐I Yeh

**Affiliations:** ^1^ Department of Medical Research Mackay Memorial Hospital Taipei Taiwan; ^2^ MacKay Junior College of Medicine, Nursing and Management Taipei Taiwan; ^3^ Division of Cardiology/Cardiovascular Center MacKay Memorial Hospital Taipei Taiwan; ^4^ Department of Medicine MacKay Medical College New Taipei City Taiwan

**Keywords:** ageing, endothelial progenitor cells, hsa‐miR‐134‐5p, peripheral blood mononuclear cells, senescence, transforming growth factor Beta 1, transforming growth factor β‐activated kinase 1‐binding protein 1

## Abstract

This research explores the role of microRNA in senescence of human endothelial progenitor cells (EPCs) induced by replication. Hsa‐miR‐134‐5p was found up‐regulated in senescent EPCs where overexpression improved angiogenic activity. Hsa‐miR‐134‐5p, which targeted transforming growth factor β‐activated kinase 1‐binding protein 1 (TAB1) gene, down‐regulated TAB1 protein, and inhibited phosphorylation of p38 mitogen‐activated protein kinase (p38) in hsa‐miR‐134‐5p‐overexpressed senescent EPCs. Treatment with siRNA specific to TAB1 (TAB1si) down‐regulated TAB1 protein and subsequently inhibited p38 activation in senescent EPCs. Treatment with TAB1si and p38 inhibitor, respectively, showed angiogenic improvement. In parallel, transforming growth factor Beta 1 (TGF‐β1) was down‐regulated in hsa‐miR‐134‐5p‐overexpressed senescent EPCs and addition of TGF‐β1 suppressed the angiogenic improvement. Analysis of peripheral blood mononuclear cells (PBMCs) disclosed expression levels of hsa‐miR‐134‐5p altered in adult life, reaching a peak before 65 years, and then falling in advanced age. Calculation of the Framingham risk score showed the score inversely correlates with the hsa‐miR‐134‐5p expression level. In summary, hsa‐miR‐134‐5p is involved in the regulation of senescence‐related change of angiogenic activity via TAB1‐p38 signalling and via TGF‐β1 reduction. Hsa‐miR‐134‐5p has a potential cellular rejuvenation effect in human senescent EPCs. Detection of human PBMC‐derived hsa‐miR‐134‐5p predicts cardiovascular risk.

## 1 INTRODUCTION

Endothelial progenitor cells (EPCs), initially identified in human peripheral blood in 1977 as CD34 antigen‐positive (CD34^+^) mononuclear cells, are bone marrow‐derived stem cells and can home to sites of vascular injury to participate in endothelial repair as well as exert angiogenesis via differentiation into endothelial cells.^1^ EPCs are recognized as a main circulating stem cell population and researched as candidate cell sources for revascularization strategies.^2^, ^3^, ^4^ Cumulative evidence suggests EPCs play an important role in defective homeostasis and vascular dysfunction. As a result, they can potentially serve as biomarkers for atherosclerotic cardiovascular diseases, which has been considered an age‐dependent condition.^5^, ^6^, ^7^, ^8^ However, several factors such as ageing and developed systemic diseases influence EPCs quantity and function, which restrict their applicability in clinical settings.^9^, ^10^, ^11^, ^12^, ^13^ In subjects with varying degrees of cardiovascular risk but no history of cardiovascular disease, a strong negative correlation was observed between the number of circulating EPCs and the participants' combined Framingham risk factor score. Furthermore, EPCs obtained from individuals at high risk for cardiovascular events exhibited higher rates of in vitro senescence compared to cells from individuals with low conventional risk factors.^14^

Ageing or senescence has been linked to a progressive decrease in the baseline circulating levels of EPCs.^15^, ^16^, ^17^ Prior studies have shown that late EPCs from elderly participants exhibit diminished angiogenic and adhesion capacities compared to those from younger cohorts. This leads to reduced capacity of EPCs to effectively repair and regenerate damaged blood vessels.^17^, ^18^, ^19^ Decline in biological function and angiogenic capacities of EPCs due to senescence or ageing may potentially lead to compromised vascular endothelial cell function. This may hinder vascular healing processes and exacerbate age‐related vascular diseases.^14^, ^20^

There has been a growing focus on the mechanisms of modulating biological functions of EPCs. MicroRNAs (miRNAs), the small single‐stranded noncoding RNAs (18–25 nucleotides), play an important role in the regulation of gene expression. Accumulating attention has been paid in recent years to the effect of changes in miRNA expression of EPCs that regulates the signalling pathway on cell function and ageing status.^21^, ^22^, ^23^, ^24^ Recently, growing data report ageing‐related miRNAs playing a key role in regulating angiogenesis in vitro, in vivo and in human study.^22^, ^25^, ^26^ MiRNAs influence replicative senescence in a sophisticated way as a result of profoundly changing the phenotype and function of proliferative progenitor cells. In this paper, we aim to uncover senescence‐related miRNAs derived from human late EPCs undergoing replicative senescence to further understand progenitor senescence and associated signalling mechanisms. This endeavour enhances understanding of the clinical impact of senescence‐related miRNA in humans from a life course—an ageing perspective.

## 2 MATERIALS AND METHODS

### 2.1 Approval of human study

Isolation of peripheral blood mononuclear cells (PBMCs) for miRNA analysis (reference number: 19MMHIS223e and 19MMHIS351e) and acquisition of late EPCs (reference number: 14MMHIS289 and 17MMHIS165) from healthy donors were approved by the Institutional Review Board of the MacKay Memorial Hospital, Taipei, Taiwan and conformed to principles outlined in the Declaration of Helsinki. Informed written consent from all participants was obtained prior to the study.

### 2.2 Study population

Eighty‐two participants from young (20–30 years), middle‐aged (31–64 years) and elderly (more than 65 years) age groups were enrolled in the study according to the following criteria: healthy adults aged 20 years or older and regardless of sex (male/female), free from infectious disease, anaemia, body weight < 50 kg, systolic blood pressure < 90 mm Hg, inpatient and pregnancy. Biochemical characters of participants are listed in Table 1. Participants aged 31 years or older were further divided into low, moderate and high risk of coronary heart disease according to Framingham risk score (https://objectivehealth.ca/clinicians/framingham/).^27^, ^28^

**TABLE 1:** Clinical characteristics of study participants.

	<31 years (*n* = 16)	≥31 to <65 years (*n* = 21)	≥65 years (*n* = 45)	*p* Value
Male/female, *n*	8/8	9/12	17/28	
Age (years), mean ± SEM	25.4 ± 0.6	41.7 ± 1.7	69.7 ± 0.5	<0.001
Body mass index (kg/m^2^), mean ± SEM	22.3 ± 0.9	24.2 ± 0.7	24.1 ± 0.5	0.221
Biochemical data, mean ± SEM				
Fasting plasma glucose (mg/dL)	88 ± 2.1	87.6 ± 1.7	105.5 ± 2.5	<0.001
Total cholesterol (mg/dL)	188.3 ± 7.8	217.9 ± 8.4	203.3 ± 6.1	0.055
HDL‐cholesterol (mg/dL)	59.9 ± 4.2	59.1 ± 4.4	57.4 ± 1.9	0.82
LDL‐cholesterol (mg/dL)	111.1 ± 8.4	135 ± 6.8	115.4 ± 4.7	0.035
Triglycerides (mg/dL)	84.8 ± 10.1	88.3 ± 10.7	104.4 ± 6.5	0.124
GPT (U/L)	20.3 ± 3	27.9 ± 4.3	27.8 ± 3.9	0.109
GOT (U/L)	20.4 ± 1	22.9 ± 1.6	25.8 ± 1.1	0.007
BUN (mg/dL)	11.6 ± 1	12.6 ± 0.6	15.1 ± 0.9	0.045
Creatinine (mg/dL)	0.8 ± 0.05	0.8 ± 0.04	0.8 ± 0.03	0.935
Health history, *n* (%)				
Alcohol drinker	5 (31.2%)	3 (14.3%)	5 (11.1%)	0.177
Current smoker	0 (0%)	0 (0%)	1 (2.2%)	>0.999
Hypertension	0 (0%)	2 (9.5%)	20 (44.4%)	<0.001
Diabetes mellitus	0 (0%)	0 (0%)	4 (8.9%)	0.304
Hyperlipidaemia	1 (6.3%)	4 (19%)	25 (55.6%)	<0.001
Drug therapy, *n* (%)				
Anti‐hypertension	0 (0%)	2 (9.5%)	20 (44.4%)	<0.001
Anti‐diabetics	0 (0%)	0 (0%)	4 (8.9%)	0.304
Anti‐hyperlipidaemia	0 (0%)	2 (9.5%)	8 (17.8%)	0.160

*Note*: Participants were divided into three groups by age. The definition of hypertension was systolic blood pressure ≥ 140 mm Hg and/or diastolic blood pressure ≥ 90 mm Hg or individuals on medication for hypertension^58^; diabetes mellitus was according to the criteria of haemoglobin A1C ≥ 6.5% or random plasma glucose ≥200 mg/dL^59^; dyslipidaemia was according to the criteria of fasting triglyceride levels ≥150 and ≤500 mg/dL, and LDL cholesterol >130 mg/dL.^60^ Data are mean ± SEM. Sample data are analysed using D'Agostino‐Pearson normality test along with either using one‐way ANOVA followed by Tukey's test for multiple group comparisons in data of age, HDL‐cholesterol and LDL‐cholesterol, or using non‐parametric tests (Kruskal Wallis test/Dunn's multiple comparison test) in data of body mass index, fasting plasma glucose, total cholesterol, triglycerides, GPT, GOT, BUN and Creatinine. In addition, data of health history and drug therapy are analysed using Fisher's exact test.

Abbreviations: BUN, blood urea nitrogen; GOT, aspartate aminotransferase; GPT, alanine aminotransferase; HDL, high‐density lipoprotein; LDL, low‐density lipoprotein.

### 2.3 Isolation of PBMCs and culture of EPCs

PBMCs were separated from healthy donors using Fecoll‐Paque™ plus (GE Healthcare). To obtain CD34^+^ cells, PBMCs were labelled with CD34 MicroBeads kit (Miltenyi Biotec) followed by purification with MACS™ Cell Separation System (Miltenyi Biotec) according to manufacturer's instrument. CD34^+^ cells were seeded onto fibronectin‐coated dishes (Corning) containing Endothelial Cell Growth Medium MV2 (PromoCell) plus 20% foetal bovine serum (FBS; Thermo Fisher Scientific) and then incubated in 5% CO_2_ at 37°C. Culture medium was replaced every 3 days. After 10–14 days, EPCs appeared as cobblestone‐like colonies and were detached by 0.05% trypsin–EDTA (Gibco) for subculture. Passages 6–10 of EPCs were defined as young group. After 10 passages, EPCs with doubling times more than twice than its own young stage were defined as senescent group.^11^, ^29^

### 2.4 Small RNA sequencing and analysis

**FIGURE 1:**
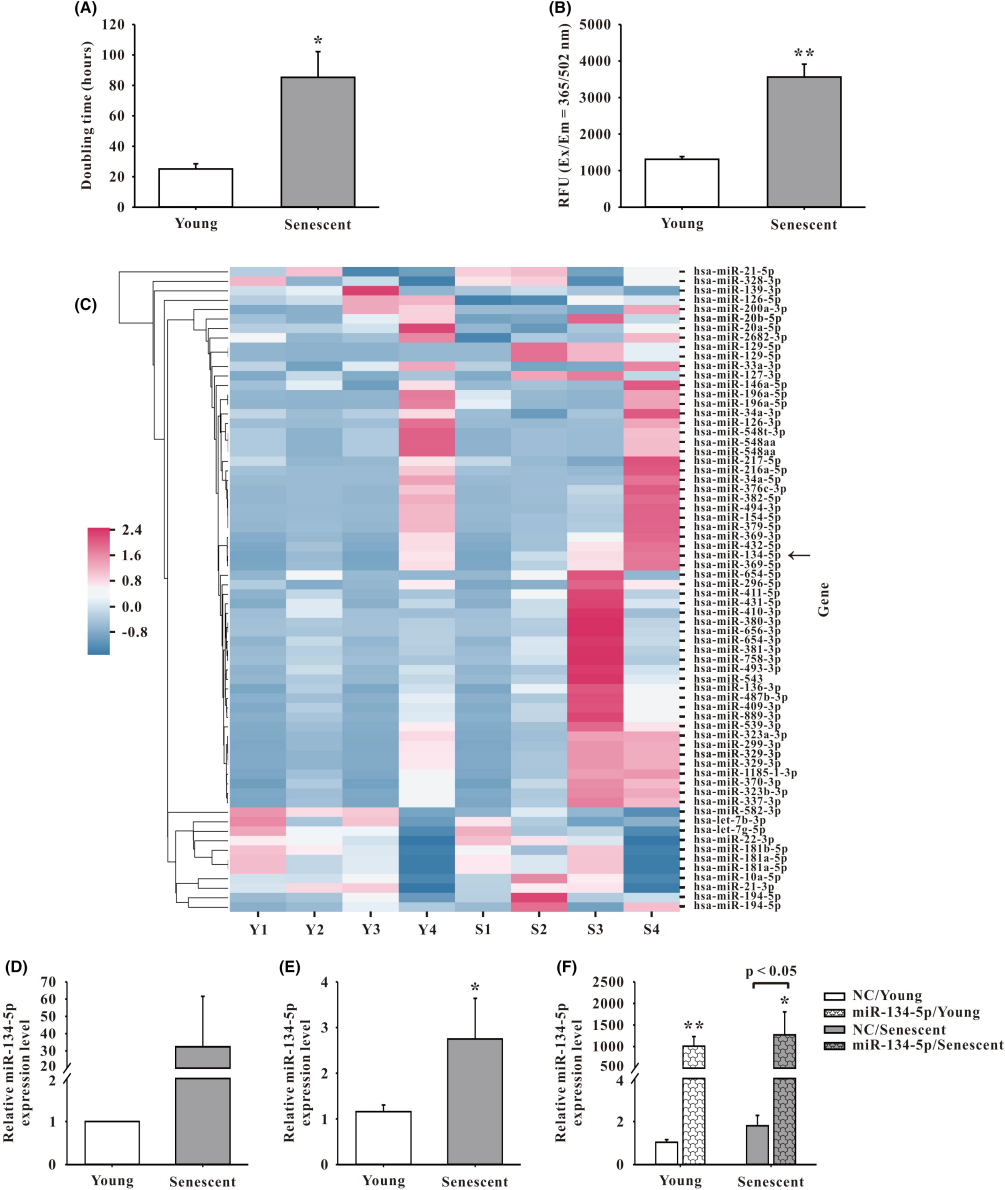
Analysis of senescence and microRNA in human endothelial progenitor cells (EPCs). (A) Doubling time of senescent EPCs versus young cells (*n* = 4), see text for details. (B) β‐galactosidase activity was significantly enhanced in senescent EPCs compared to young cells (*n* = 3). (C) Screening of senescence‐associated microRNA in human EPCs using small RNA sequencing and analysis. Heat map of miRNA expression data obtained from four human donors. Relative miRNA expression was depicted according to the colour scale shown. Red indicates up‐regulation and grey indicates down‐regulation. The young EPCs (Y1–Y4) represented three repeats for each donor's EPCs along with the data of the corresponding senescent EPCs (S1–S4). Arrow, hsa‐miR‐134‐5p. (D) Small RNA analysis showed expression of hsa‐miR‐134‐5p was increased by more than 30‐folds in senescent EPCs compared to young cells (*n* = 4). (E) Quantitative real‐time‐PCR (qRT‐PCR) showed an increased expression of hsa‐miR‐134‐5p in the senescent EPCs, compared to young EPCs (*n* = 13). (F) Enhanced expression of hsa‐miR‐134‐5p was detected using qRT‐PCR in young and senescent EPCs transfected with miR‐134‐5p mimics, compared to corresponding NC (*n* = 5). Also note that, the difference between control senescent group (NC/Senescent) and miR‐134‐5p‐overexpressed senescent group (miR‐134‐5p/Senescent) was indicated. Data are mean ± SEM and analysed using unpaired Student's *t*‐test for comparison between groups. *p < 0.05, **p < 0.01 versus corresponding control. Em, emission; Ex, excitation; NC, negative control; RUF, relative fluorescence unit.

Total RNA was extracted from human EPCs by Trizol reagent® (Invitrogen) and quantified by OD260/OD280 absorbance ratio under spectrophotometer (Nanodrop Technology). Quality control of total RNA was performed using 2100 Bioanalyzer system (Agilent Technology). RNA samples were commissioned to Welgene Biotechnology. Co., Ltd. (Taipei, Taiwan) for library preparation using QIAseq miRNA Library Kit (QIAGEN) and sequenced under Illumina instrument (75‐cycle single‐end read, 75SE). Sequencing data was processed by Illumina software (BCL2FASTQ v2.20). The miRNA expression levels of young and senescent EPCs were compared with reads per million (RPM). The difference of miRNA expression level between two groups more than twofolds were considered as significance (Figure 1C).^25^, ^30^

### 2.5 MiRNA transfection

EPCs (7 × 10^4^) were seeded onto 3.5 cm dishes. After 24 h of culture, hsa‐miR‐134‐5p mimic (Ambion, order number: 4464066) and miRNA mimic negative control (NC) (Ambion, order number: 4464058; Figures 1F, 2A–C, 3A–C, 4G–I) were respectively transfected to EPCs at 25 nM using Lipofectamine 3000 (Thermo Fisher Scientific) according to manufacturer's instrument. After 5.5 h of transfection, medium containing transfection reagent was replaced with MV2 basal medium plus 20% FBS. After next 48 h of culture, hsa‐miR‐134‐5p mimic‐transfected EPCs were used for following experiments.^11^, ^25^

**FIGURE 2:**
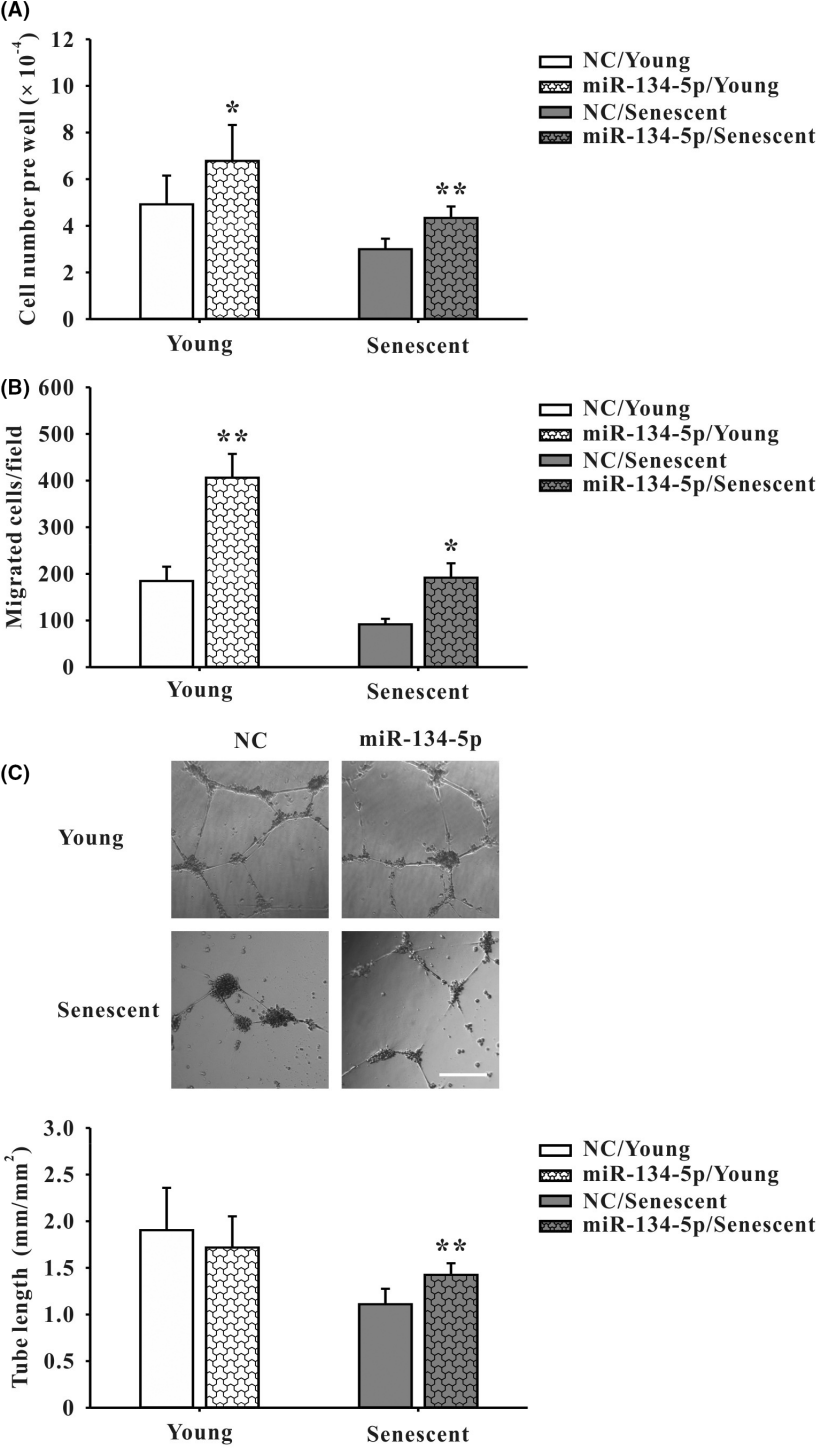
Measurement of angiogenic activities 48 h after hsa‐miR‐134‐5p mimics treatment in human EPCs. In the foetal bovine serum (FBS)‐containing medium MV2 (A–C), compared to corresponding NC, transfection with miR‐134‐5p mimics increased (A) cell number detected by cell counting in both young and senescent groups (*n* = 7), (B) cell migration using Boyden Chamber assay in both young and senescent groups (*n* = 5) and (C) tube formation using Matrigel in senescent group but not young group (*n* = 5). Representative micrographs are shown in (C). Scale bar: 300 μm. Data are mean ± SEM and analysed using unpaired Student's *t*‐test for comparison between groups. *p < 0.05, **p < 0.01 versus corresponding control. NC, negative control. Scale bar, 300 μm in image C.

**FIGURE 3:**
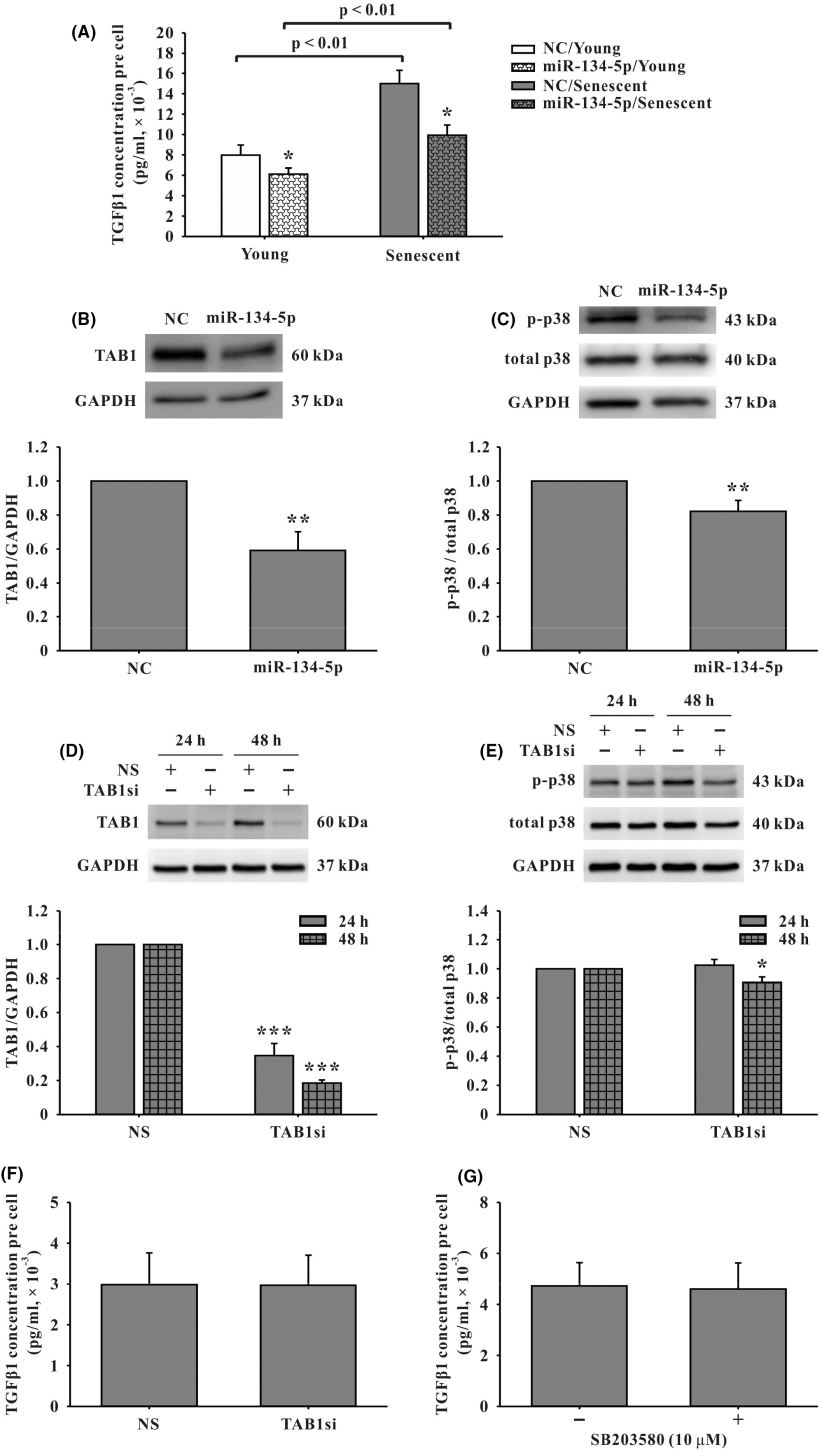
Evaluation of signalling regulation of hsa‐miR‐134‐5p in human EPCs by enzyme‐linked immunosorbent assay (ELISA) and western blot analysis. In miR‐134‐5p‐overexpressed EPCs, (A) transforming growth factor Beta 1 (TGF‐β1) level in the supernatant normalized by cell number was respectively reduced in young and senescent cells, compared to miRNA mimic negative control (NC) group (*n* = 4). Also note, the difference between young and senescent groups was indicated. The findings showed that, when performing a comparison between groups with or without overexpression of miR‐134‐5p, the expression level of TGF‐β1 was higher in senescent than young EPCs. Additionally, in senescent EPCs, (B) the TGF‐β1‐associated protein, transforming growth factor β‐activated kinase 1‐binding protein 1 (TAB1) (*n* = 5) and (C) phosphorylated p38 mitogen‐activated protein kinase (p‐p38, *n* = 3) were down‐regulated compared to NC. (D) After senescent EPCs were treated with siRNA specific to TAB1 (TAB1si) for 24 h (h) and 48 h, TAB1 was suppressed (*n* = 7) and, subsequently, (E) p‐p38 was down‐regulated at the time point of 48 h after TAB1si treatment compared to non‐sense siRNA (NS) (*n* = 6). (F) and (G) When TAB1si or p38 mitogen‐activated protein kinase inhibitor SB203580 was added to senescent EPCs, TGF‐β1 level in the supernatant normalized by cell number showed minimal change between treatment group and its corresponding control group/(*n* = 4). Data are mean ± SEM and analysed using unpaired Student's *t*‐test for comparison between groups. *p < 0.05, **p < 0.01 and ***p < 0.001 versus corresponding control.

**FIGURE 4:**
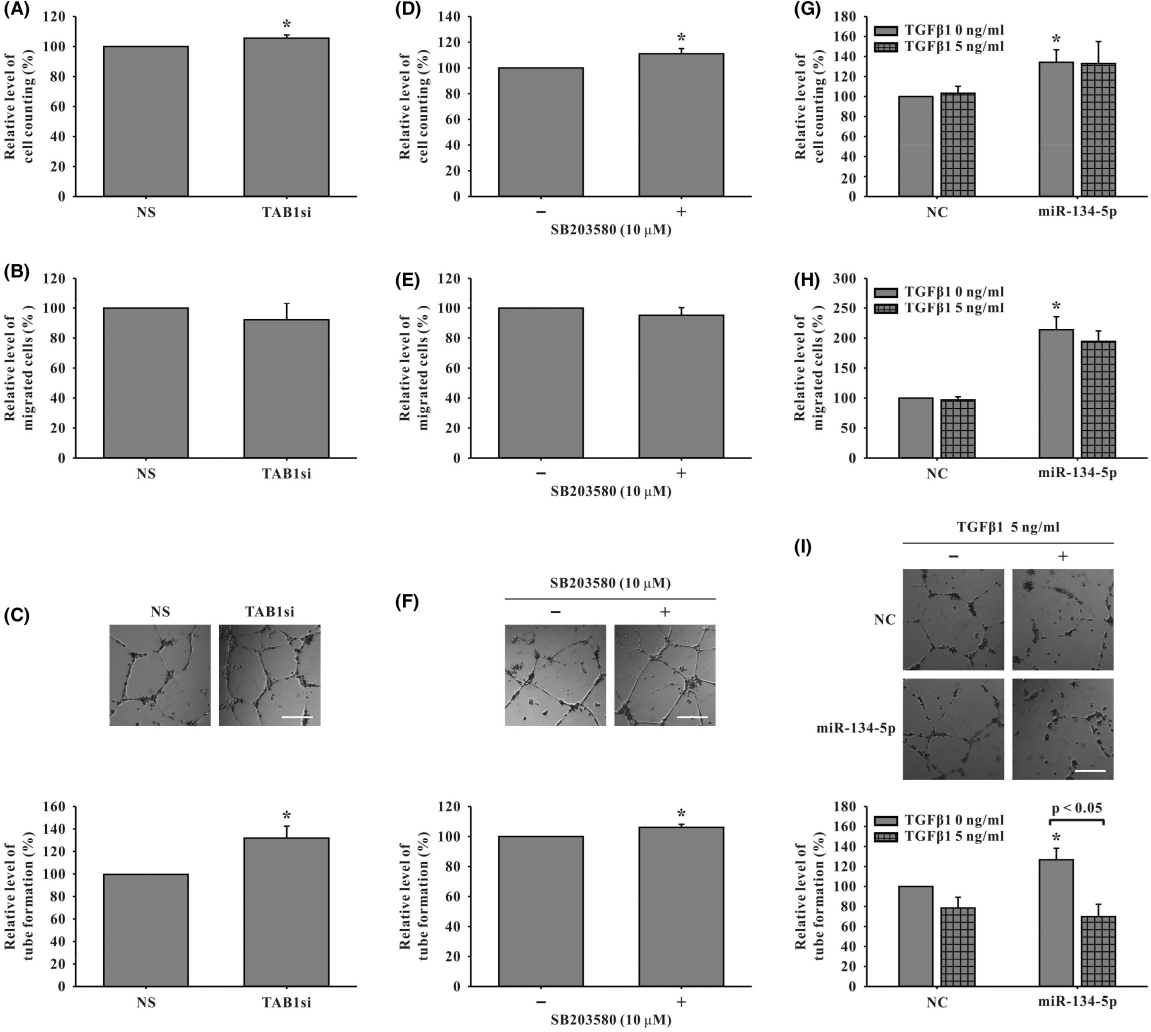
Angiogenic activities of senescent human EPCs after treatment with siRNA specific to TAB1 (TAB1si)/p38 inhibitor (SB203580, 10 μM) and transforming growth factor Beta 1 (TGF‐β1, 5 ng/mL). In TAB1si‐treated EPCs, improved cellular activities of (A) cell number detected by cell counting (*n* = 5) and (C) tube formation using Matrigel (*n* = 3) were noted but not found in (B) migration using Boyden Chamber assay (*n* = 4). Similarly, after treatment with SB203580, (D) cell number (*n* = 5) and (F) tube formation (*n* = 4) were improved but (E) migration (*n* = 6) was not improved. In senescent EPCs with improved angiogenic activities after overexpression of miR‐134‐5p, TGF‐β1 treatment did not affect (G) cell number (*n* = 4) and (H) migration (*n* = 5), but (I) tube formation (*n* = 6) was inhibited, compared to corresponding miR‐134‐5p‐overexpressed EPC group without TGF‐β1 treatment. Representative micrographs are shown in (C), (F) and (I). Scale bar: 300 μm. Data are mean ± SEM. Statistical significance was analysed using unpaired Student's *t*‐test for comparison between two groups in (A)–(F) or using ANOVA with Tukey's post hoc test for multiple group comparisons in (G)–(I). *p < 0.05 versus corresponding control. Abbreviations are the same as in Figure 3.

### 2.6 SiRNA transfection

EPCs (7 × 10^4^) were seeded onto 3.5 cm dishes. After 24 h of culture, human transforming growth factor β‐activated kinase 1‐binding protein 1 (TAB1) siRNA and non‐sense siRNA (NS) (all from Dharmacon; Figures 3D–F and 4A–C) were respectively transfected to EPCs using RNAiMAX reagent (Thermo Fisher Scientific) at 20 nM for 24 h according to manufacturer's instrument. After a 24‐h culture, siRNA transfected EPCs were collected for western blotting and angiogenic activity assays.^29^

### 2.7 Drug treatment

To evaluate the effects of transforming growth factor Beta 1 (TGF‐β1) on angiogenic activities of miR‐134‐5p mimic‐transfected EPCs, EPCs were cultured in 20% FBS containing MV2 basal medium with or without 5 ng/mL of TGF‐β1 (R&D Systems) for 48 h after transfection.^31^ Angiogenic activity assays were conducted in TGF‐β1‐treated EPCs. To evaluate whether p38 mitogen‐activated protein kinase (p38) activation related to angiogenic activities of EPCs, EPCs were cultured in 2% FBS containing MV2 basal medium with or without 10 μM p38 inhibitor (SB203580; R&D Systems) for 1 h. After next 24 h of culture, angiogenic activity assays and western blotting were conducted in SB203580‐treated EPCs.

### 2.8 Senescence‐associated β‐galactosidase activity analysis

SA‐β‐Gal activity of EPCs was evaluated using Cellular Senescence Assay Kit (Cell Biolabs) according to the manufacturer's instruction. EPCs were seeded on a 24‐well plate (1 × 10^4^ per well) and cultured for 48 h. EPCs were lysed in 80 μL of lysis buffer and then incubated with equal volume of assay buffer containing SA‐β‐Gal substrate for 2 h at 37°C. After stopping the reaction with stop solution, fluorescence was read at 365 nm (excitation)/502 nm (emission).

### 2.9 Quantitative real‐time PCR (qRT‐PCR)

Total RNA was extracted from EPCs by Quick‐RNA MiniPrep Plus kit (Zymo Research). Reverse transcription was performed by Mir‐X™ miRNA First‐Strand Synthesis kit (Takara). For real‐time PCR, cDNA was amplified by iTaq Universal SYBR Green Supermix (BIO‐RAD) in the presence of primers for miR‐134‐5p (GeneCopoeia, HmiRQP0168) or U6 (from Mir‐X™ miRNA First‐Strand Synthesis kit) under StepOnePlus™ Real‐Time PCR Systems (Applied Biosystems). Relative miRNA expression levels were normalized with the corresponding levels of U6 and analysed by delta–delta CT methods.^25^

### 2.10 Human cytokine array and ELISA

The supernatant of EPC culture was collected at 48 h post‐transfection of miR‐134‐5p mimic or mimic negative control. The composition of EPC supernatant was analysed by Human Cytokine Array C1000 (RayBiotech) according to the manufacturer's instruction. Density of blots were analysed by VisionWorks software (Analytik Jena). For total protein measurement, the concentrations of TGF‐β1, interleukin‐7 (IL‐7) and tumour necrosis factor alfa (TNF‐α) in supernatant were determined by ELISA kit (R&D systems) according to the manufacturer's instruction. The absorbance was read at 450 nm and corrected with 540 nm.

### 2.11 Western blot

EPCs were lysed in SB‐20 buffer (0.69 mol/L SDS, 10 mmol/L EDTA, 100 mmol/L Tris–HCL, pH 6.8) containing protease and phosphatase inhibitor (Roche).^29^ Cell lysate (25 μg) was loaded into 12% SDS‐polyacrylamide gels for electrophoresis and transblotted onto PVDF membranes (Merck Millipore). The blots were blocked with 0.1 g/mL BSA for 1 h at room temperature and subsequently incubated with primary antibodies specific to TAB1, phosphorylated p38 (Thr180/Tyr182) and total p38 (all from Cell Signalling) with the dilution 1:1000 at 4°C for 16 h.^29^ The blotting membranes were then incubated with HRP‐conjugated secondary antibodies (1:10,000, Jackson ImmunoResearch) for 1 h at room temperature. To normalize expression level, blots were stripped with Ultra stripping reagent (Bionovas) at room temperature for 10 min and then incubated with anti‐GAPDH (1:1000, Thermo Fisher Scientific) as an internal control.^32^ The blot images were photographed by UVP ChemiDoc‐It 815 Image system (Ultra Violet Products, Ltd). Band density of blot was calculated by VisionWorks software.

### 2.12 Angiogenic activity assay

Cell survival, migration and tube formation assays were conducted in EPCs to evaluate angiogenic activities. For cell survival analysis, viable cells were evaluated by trypan blue (Bio‐Rad) staining followed by counting number under TC10™ automated cell counter (Bio‐Rad).

For cellular migration analysis, Boyden chamber (8 μm pore size) assay was conducted in EPCs. Cells (3 × 10^4^) in 100 μL of basal MV2 medium were seeded on upper chamber, and 600 μL of basal MV2 medium plus 2% FBS was put in lower chamber. After 37°C incubation for 4 h, the cells were fixed with −20°C methanol for 5 min and stained with 18.7 mM bisbenzimide H33258 (Sigma‐Aldrich) for 15 min at room temperature. EPCs migrated to the lower side of the filter was recorded by fluorescence microscopy (Leica) and counted by QWin (Leica) software.^33^, ^34^

To evaluate tube formation capacity of EPCs, Matrigel (corning) was added in a 24‐well plate (200 μL/each well) and polymerized at 37°C for 30 min. Thereafter, 3 × 10^4^ EPCs suspended in 1 mL of MV2 basal medium plus 2% FBS were seeded to Matrigel‐coated wells and incubated at 37°C in a 5% CO_2_ incubator. After 24 h of culture, tube formation was photographed with a Leica camera (50× magnification). Tube length was measured in randomly fields from each well by QWIN software (Leica).^33^, ^34^

### 2.13 Doubling time of cell proliferation

EPCs were seeded on 24‐well plates (1 × 10^4^ per well). Doubling time of EPC proliferation was evaluated by viable cell count at 0, 24, 48, 72 h after culture as described above. Thereafter, cell numbers were entered to the URL (https://www.doubling‐time.com/compute.php) to calculate proliferative doubling time.

### 2.14 Statistical analysis

For in vitro studies, results were presented as mean ± SEM. GraphPad Prism version 9.0 was used to perform statistical analysis. Student's *t‐*test was used to compare between two groups, and ANOVA test was used to compare more than three groups. For clinical study, the results of clinical data were presented as mean ± SD. D'Agostino and Pearson test was used for normality of distribution analysis. When normality test was passed, Student's *t*‐test was conducted to compare between two groups, and ANOVA with Tukey's post hoc test was used to compare more than three groups. When normality test was not passed, Kruskal–Wallis test and Dunn's multiple comparison tests or Spearman's rank correlation coefficient were selected for nonparametric statistical analysis. A *p* < 0.05, was considered statistically significant.

## 3 RESULTS

### 3.1 Identification of senescence‐associated microRNA in human EPCs

In this study, senescence of human EPCs was defined as cells with an increase of doubling time more than twofolds than that of young cells (Figure 1A). SA‐β‐Gal assay showed that the absorbance increased by more than 100% in the senescent cells (Figure 1B). The human late EPCs from four donors were analysed using small RNA sequencing. Candidate microRNAs central to senescence regulation were selected when the expression levels in the senescent EPCs were more than 20 times higher compared to the young EPCs. A total of seven microRNAs fulfilled the criteria, including hsa‐miR‐127‐3p, hsa‐miR‐411‐5p, hsa‐miR‐134‐5p, hsa‐miR‐299‐3p, hsa‐miR‐409‐3p, hsa‐miR‐758‐3p and hsa‐miR‐889‐3p. Thereafter, the significantly higher expression levels were further confirmed by qRT‐PCR, followed by overexpression experiments of the microRNAs in the EPCs. The results showed that only hsa‐miR‐409‐3p and hsa‐miR‐134‐5p had significant cellular effects (see details in Section 3.2 for cellular effects of hsa‐miR‐134‐5p). Since the miR‐409‐3p story had been reported,^25^ in this study, we focused on the role of miR‐134‐5p in human EPC senescence. The results of small RNA sequencing showed that the expression of hsa‐miR‐134‐5p was enhanced in senescent EPCs, compared to young EPCs (Figure 1C,D). The result was further confirmed using qRT‐PCR analysis (Figure 1E). Compared to corresponding NC, transfection using lipofectamine 3000 with miR‐134‐5p mimics significantly up‐regulated the expression of miR‐134‐5p in both young and senescent EPCs (Figure 1F).

### 3.2 Evaluation of hsa‐miR‐134‐5p‐mediated angiogenic activity in human EPCs in vitro

Concerning the cellular effects of hsa‐miR‐134‐5p, overexpression of miR‐134‐5p in young and senescent EPCs was performed in the FBS‐containing medium MV2. Enhanced angiogenic activity was noted in cell number (Figure 2A) and migration (Figure 2B) of both young and senescent cells. In contrast, tube formation activity was only enhanced in senescent group (Figure 2C), compared to corresponding NC.

### 3.3 Exploration of hsa‐miR‐134‐5p‐targeted signalling pathway in human EPCs

To investigate the biological target and regulatory signalling of hsa‐miR‐134‐5p in human EPCs, since there is no EPC‐specific cytokine array commercially available, a membrane‐based antibody array (Human Cytokine Antibody Array C1000) for the initial determination of relative levels of selected human cytokines and chemokines was utilized. The supernatant derived from miR‐134‐5p‐overexpressed senescent EPCs was added to the antibody array. We defined that a more than 50% change in densitometry analysis was considered significant. The results showed a significant change in the total TGF‐β1, IL‐7 and TNF‐α protein levels, compared to control cells without miR‐134‐5p‐overexpression (Figure S1A). Subsequent ELISA showed a significant down‐regulation of TGF‐β1 protein level normalized by cell number, compared to the corresponding negative control (NC) group in young and senescent EPCs (Figure 3A). In contrast to TGF‐β1, ELISA assay revealed no changes in IL‐7 levels, while it failed to detect TNF‐α. The membranes of protein array and ELISA of IL‐7 were shown in the Figure S1A,B. Since our research focused on the role of miR‐134‐5p in EPC senescence, the subsequent experiments were only conducted in senescent EPCs. Thereafter, both the TargetScan and miRanda web servers identified a possible target gene of hsa‐miR‐134‐5p, TAB1 gene, which was associated with TGF‐β1 and correlated to the finding shown in the literature^35^ that the seed sequences of hsa‐miR‐134‐5p and its predicted binding site was located in the 3′‐UTR (untranslated region) of TAB1 gene. Furthermore, in the literature, p38 kinases had been linked to processes related to cell cycle, cell development and senescence. Their phosphorylation can be induced through binding to TAB1.^36^, ^37^ Therefore, the miR‐134‐5p‐targeted signalling pathway in senescent human EPCs was then investigated by western blot analysis. The results showed that, in miR‐134‐5p‐overexpressed senescent EPCs, both TAB1 (Figure 3B) and phosphorylated p38 mitogen‐activated protein kinase (p‐p38) were down‐regulated at protein level (Figure 3C) compared to corresponding NC. In senescent EPCs treated with siRNA specific to TAB1 (TAB1si) (Figure 3D), compared to corresponding NS, p‐p38 was down‐regulated at the time point of 48 h after the TAB1si treatment (Figure 3E). In addition, after the senescent EPCs were treated with TAB1si or p38 mitogen‐activated protein kinase inhibitor SB203580, there were minimal changes of TGF‐β1 level in the supernatant of each treatment group normalized by cell number, in comparison with corresponding control (Figure 1F,G).

These findings revealed that in human senescent EPCs, TGF‐β1 was regulated by miR‐134‐5p and TAB1‐p38 signalling was regulated by miR‐134‐5p, but TGF‐β1 was not the down‐stream effector of TAB1‐p38 signalling.

### 3.4 Measurement of angiogenic activity in human senescent EPCs treated with siTAB1/p38 inhibitor (SB203580) or TGF‐β1

To validate that the TAB1‐p38 signalling pathway was involved in the hsa‐miR‐134‐5p‐mediated angiogenic activities in human senescent EPCs, activities were evaluated after treatment with either TAB1si or p38 inhibitor (SB203580, 10 μM). Results showed improved cellular activities including cell number and tube formation after TAB1 protein treatment was suppressed in siTAB1‐treated EPCs (Figure 4A,C) but not migration (Figure 4B). Similar improved angiogenic activities were found in p38 inhibitor (SB203580)‐treated senescent EPCs (Figure 4D–F). Furthermore, similar to the data shown in Figure 2A–C, all three cellular activities were enhanced in miR‐134‐5p‐overexpressed senescent EPCs (Figure 4G–I). Further adding TGF‐β1 (5 ng/mL) to miR‐134‐5p‐overexpressed senescent EPCs impeded tube formation (Figure 4I) but minimal inhibition was found in cell number and migration (Figure 4G,H).

As a result, by targeting TAB1‐p38 signalling and affecting TGF‐β1, hsa‐miR‐134‐5p mediated the improvement of all three angiogenic activities in human senescent EPCs with differential roles of TAB1, p38 and TGF‐β1 in each of cell survival, migration and tube formation.

### 3.5 Age‐dependent expression levels of hsa‐miR‐134‐5p in human PBMCs

To evaluate the clinical implications of hsa‐miR‐134‐5p, PBMCs were collected from 82 donors and the hsa‐miR‐134‐5p expression level was determined using qRT‐PCR. Since the Framingham risk score is applicable to individuals aged 30 years or older and the generally accepted definition of old age is 65 years or more, we categorized the donors into three groups: group 1: <31 years, group 2: 31–64 years and group 3: ≥65 years. Characteristics of the participating donors are shown in Table 1. There were differences among groups in age, fasting plasma glucose, LDL‐cholesterol, aspartate aminotransferase (GOT), blood urea nitrogen (BUN), percentages of patients with hypertension and hyperlipidaemia, as well as those taking hypertension medication (Table 1).

**FIGURE 5:**
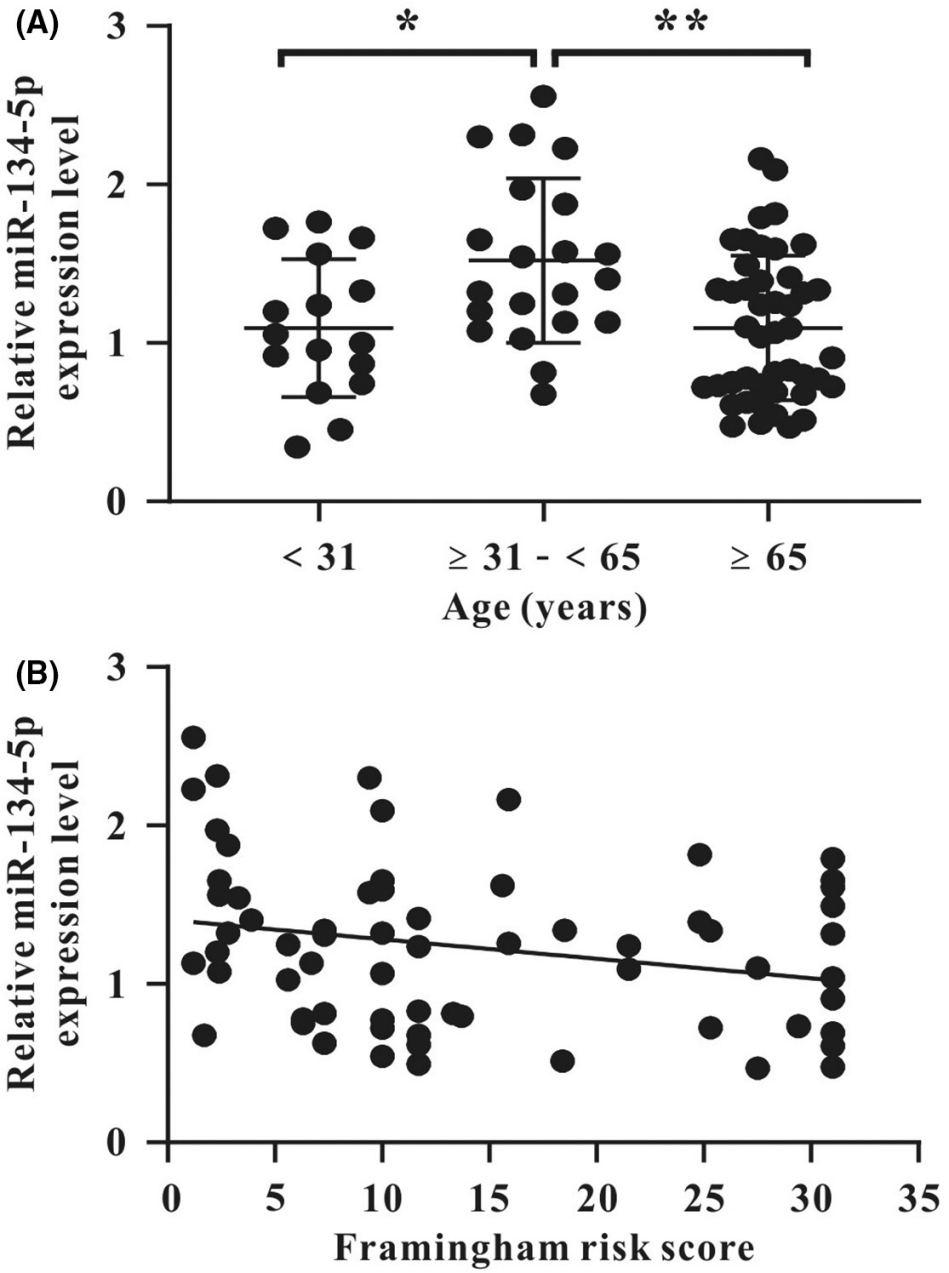
Dot plots analysis of hsa‐miR‐134‐5p transcript level in human peripheral blood mononuclear cells (PBMCs) from participants using quantitative real‐time PCR (qRT‐PCR). (A) In all donors along the age, compared to the group less than 31 years (*n* = 16), the expression level initially increased in the group aged 31 to less than 65 years (*n* = 21), and then decreased in the group aged 65 years or more (*n* = 45). (B) In the participants aged 31 years or more (*n* = 66), as the Framingham risk score increased, the expression level decreased (*r* = −0.2543, *p* = 0.0393) by Spearman's rank correlation coefficient. Data are shown as mean ± SD in (A). Statistical significance was analysed using D'Agostino‐Pearson normality test along with using one‐way ANOVA followed by Tukey's test for multiple group comparisons in (A). *p < 0.05, **p < 0.01 versus corresponding control.

With the cut‐off age at 65 years, the average hsa‐miR‐134‐5p expression level in PBMCs of donors aged 65 years or over was significantly decreased compared to the level of the control donors (Figure 5A). However, along the adult life, the miR‐134‐5p expression level was significantly enhanced in group 2, respectively, compared to group 1 and group 3 (Figure 5A). Importantly, when Framingham risk score with conventional risk factors including gender, age, systolic BP, on treatment for hypertension, total cholesterol, HDL/LDL cholesterol, cigarette smoking, diabetes and family history of CAD (https://objectivehealth.ca/clinicians/framingham/)^27^, ^28^ was utilized to investigate the correlation between expression level of miR‐134‐5p and cardiovascular risk, the risk score increased as the expression level decreased (*r* = −0.2543, *p* = 0.0393) by Spearman's rank correlation coefficient test (Figure 5B).

**FIGURE 6:**
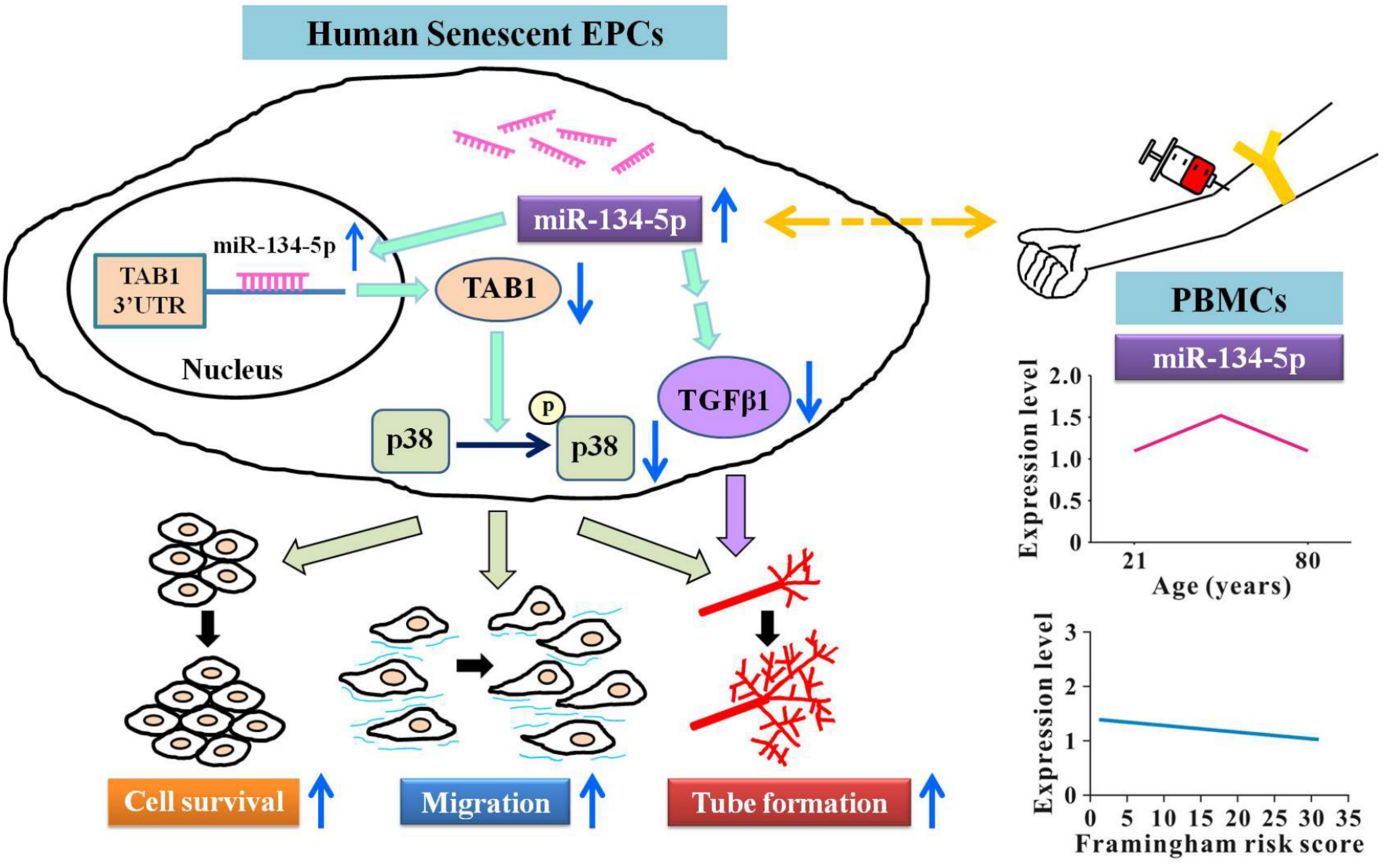
Signalling regulation of hsa‐miR‐134‐5p in human senescent EPCs and its clinical implication. In human EPCs cultured from PBMCs, miR‐134‐5p in the senescent cells, mainly targeting TAB1 gene, is up‐regulated and such a change results in improvement of angiogenetic activity via TAB1/p38 signalling. Moreover, up‐regulated miR‐134‐5p in senescent EPCs is linked to TGF‐β1 modulation to promote tube formation activity. In PBMCs, the expression level of hsa‐miR‐134‐5p has an initial increase after 31 years, and a subsequent decrease after 65 years. Moreover, along the increase of Framingham risk score, the expression level of hsa‐miR‐134‐5p decreases. Hsa‐miR‐134‐5p presents its potential anti‐ageing character in the human ageing process and acts as a pro‐angiogenesis modulator. Abbreviations are as in Figure 3.

Novel findings of regulation of hsa‐miR‐134‐5p in the senescence of human EPCs derived from PBMCs, the relevant signalling pathways, potentially epigenetic modification of angiogenic activities and clinical implications are summarized in Figure 6.

## 4 DISCUSSION

EPCs are mononuclear cells that circulate in the blood and are currently being researched as candidate cell sources for neovascularization strategies. However, substantial age‐related decline in EPC number and function limits clinical application.^9^, ^10^, ^11^, ^38^, ^39^ To investigate cell activities of young versus senescent EPCs in vitro regarding miRNA expression, the in vitro part of this human study induced senescence of EPCs to a modest level using replication of certain passages. Senescent EPCs were defined to have an increase in cell doubling time more than twofolds of the young cells, accompanied by an increase of up to 200% in acidic β‐galactosidase activity.^25^ Exploration of senescence‐related microRNA using this replication‐induced senescence model showed that hsa‐miR‐134‐5p contributed to the angiogenic improvement of senescent EPCs via TAB1/p38 signalling and TGF‐β1‐related pathways. In parallel, investigation of the human PBMCs demonstrated that hsa‐miR‐134‐5p was initially raised and then down‐regulated along the adult life. Hsa‐miR‐134‐5p was first identified by cloning studies of mouse^40^ and its expression then verified in human embryonic stem cells.^41^ Growing evidence suggests epigenetic modifications by regulation of microRNA are linked to EPCs and angiogenesis.^42^, ^43^, ^44^, ^45^, ^46^, ^47^ For example, miR‐9‐5p was found to promote migration, invasion and angiogenesis of EPCs via activating PI3K/Akt/autophagy pathway.^48^ MiR‐205 inhibition was reported to sufficiently enhance proliferative, migratory and angiogenic activity of late EPCs in vitro through mechanisms linked to NOTCH2 targeting.^49^ MiR‐126 was shown to promote the functions of EPCs under hypoxic conditions by negatively targeting PIK3R2.^50^ However, whether these microRNAs were up‐regulated by senescence was unknown. In contrast to previous findings, our report shows pioneering evidence of differential expression of a microRNA, hsa‐miR‐134‐5p, in young versus senescent EPCs, and its contributions to angiogenic improvement of senescent EPCs with cardiovascular risk‐related expression level in adult life.

In literature, no research examined the regulatory link between hsa‐miR‐134‐5p and human EPCs or hsa‐miR‐134‐5p‐associated improvement of angiogenesis in senescent EPCs. One may wonder about the rationale of using very strong overexpression in this study to mimic the functional consequence of about twofolds of miR‐134‐5p difference between young and senescent EPCs. Since cell culture experiments only allowed hours or days to evaluate the effects of microRNA, considering years of time for EPCs to face the up‐regulation of miR‐134‐5p during the ageing process, we think it is reasonable using very strong miR‐134‐5p overexpression to represent the long‐term effects, similar to previous reports in various research fields,^6^, ^42^, ^47^, ^48^, ^49^, ^50^, ^51^, ^52^, ^53^ in which the microRNA expression fold changes between cells and their control groups usually ranged from twofold to 20‐folds, while in the forced expression experiments in vitro the microRNA expression levels after forced expression could be as high as more than a few 100‐fold. In our study, TGF‐β1 protein level was proved to be down‐regulated in senescent EPCs‐derived supernatant by ELISA. MicroRNA target prediction by TargetScan and miRanda subsequently demonstrated TAB1 as a possible biological target of hsa‐miR‐134‐5p. This finding correlated to a previous report of human hepatic stellate cells, in which its predicted binding site was located in the 3′‐UTR of TAB1 gene.^35^ Therefore, no experiment was repeated in our study to examine hsa‐miR‐134‐5p and its binding site in the target gene (TAB1). According to the literature, activation of p38 kinase by both stress and mitogenic stimuli in a cell‐dependent manner was associated with cell cycle, cell survival and senescence in which, to the activation of p38 by upstream kinases, there was a MAPK kinases‐independent mechanism of activation involving TAB1.^36^, ^37^, ^51^, ^54^ In our study, TAB1 and p‐p38 were both found down‐regulated in miR‐134‐5p‐overexpressed senescent EPCs. Forty‐eight hours after adding TAB1si at the same cellular conditions, p‐p38 was down‐regulated and hence proved to be the downstream signalling molecule of TAB1. Therefore, hsa‐miR‐134‐5p regulated senescence of human EPCs at least by targeting TAB1‐p38 signalling. Moreover, concerning the down‐regulated TGF‐β1 level in the supernatant of miR‐134‐5p‐overexpressed senescent EPCs found at the beginning of this study, we eventually found that TGF‐β1 protein level was not significantly altered at the same cellular conditions after adding TAB1si or the p38 inhibitor, implying that miR‐134‐5p affects senescent EPCs via TGF‐β1 regulation independent of TAB1‐p38 signalling.

So far, there has been no clear evidence about hsa‐miR‐134‐5p‐related signalling pathway in angiogenesis of human senescent EPCs. In this study, remarkably, enhancements of cell survival/tube formation except migration were found in TAB1si‐ or p38 inhibitor‐treated senescent EPCs, confirming the miR‐134‐5p‐targeting TAB1‐p38 signalling in senescent EPCs. Moreover, adding TGF‐β1 protein significantly inhibited improvement of tube formation in response to overexpression of miR‐134‐5p with minimal effects in cell survival and migration activities, supporting the involvement of TGF‐β1 in miR‐134‐5p‐regulating senescent EPCs. Together, TAB1‐p38 pathway and TGF‐β1 regulation, respectively, had specific actions on different parts of cell activities, resulting in enhancing angiogenic activities governed by hsa‐miR‐134‐5p in senescent EPCs.

Gene regulation is the main role of miRNAs in the human body by enhancing the degradation of messenger RNAs, thereby impacting translation.^55^, ^56^ Potential roles of miRNAs include acting as a reliable biomarker and/or therapeutic target for clinical application. At the beginning of our study, we sought to find a key microRNA to rejuvenate human senescent EPCs. According to the literature, ageing‐associated miR‐217 aggravates atherosclerosis and promotes cardiovascular dysfunction, acting as a biomarker of vascular ageing and cardiovascular risk.^6^ Up‐regulation of miR‐548j‐5p promotes angiogenesis in ischaemic tissue and may represent a novel therapeutic target for peripheral artery disease.^52^ More recently, circulating miR‐28‐5p and let‐7d‐5p were identified as potential biomarkers of the premature ageing in individuals with Down syndrome before the age of 50 years, as their levels declined earlier compared to non‐trisomic physiological ageing.^53^ Circulating miR‐29b decreased in response to sarcopenia in older Chinese patients with cardiovascular risk factors and could be considered as a possible biomarker for sarcopenia.^57^ In our previous research, hsa‐miR‐409‐3p was up‐regulated in senescent EPCs, acting as a negative modulator of angiogenesis and could be a potential biomarker for human ageing.^25^ In this study, since TAB1‐p38 signallings and TGF‐β1 regulation were first linked to hsa‐miR‐134‐5p‐regulated angiogenic enhancement in senescent EPCs, we investigated the clinical impact of miR‐134‐5p. Owing to the difficulties of accumulating enough human EPCs from PBMCs for microRNA analysis, we examined PBMCs instead of EPCs. Our in vitro experiments showed angiogenic improvement in replication‐induced senescent EPCs overexpressing miR‐134‐5p. One possibility was that miR‐134‐5p could be an anti‐senescence miRNA in human middle life, when the microRNA was up‐regulated to compensate the ageing‐related decline in EPC function. However, the compensation was exhausted as the ageing process progressed, and the microRNA level declined in advanced age. In addition, when the Framingham risk score, only applicable to individuals older than 30 years, was applied in the groups more than 30 years old, the negative correlation between expression level of miR‐134‐5p and cardiovascular risk was illustrated.

Limitations exist in this research. First, signalling mechanisms observed in human EPCs may not occur in PBMCs, where the clinical implication of hsa‐miR‐134‐5p in ageing stood for. Second, blood donors in our study population were mainly volunteers and staff in the hospital, who may be healthier than the general population. Third, the number of participants was relatively small and human blood samples of all experiments used in this study were derived from Taiwanese. Additionally, the hsa‐miR‐134‐5p‐regulated cellular effect was not investigated in human young EPCs, and the rejuvenation effect was not further applied in animal studies. Meanwhile, the very strong overexpression of hsa‐miR‐134‐5p may overestimate the effects in human life. Future research is required to clarify these issues.

In summary, our investigation indicates that hsa‐miR‐134‐5p is a remarkable epigenetic modulator and could be a microRNA with potential cellular rejuvenation effects in human senescent EPCs. MiR‐134‐5p was involved in the regulation of anti‐senescence signalling pathways via TAB1‐p38 signalling as well as via TGF‐β1 regulation. The human PBMC study pioneeringly showed the expression level of miR‐134‐5p altered in human adult life, reaching a peak before 65 years and then dropping in advanced age (≥65 years). These findings highlighted the clinical potential of detecting human PBMC‐derived hsa‐miR‐134‐5p for biological ageing. This is the first report linking microRNA in human PBMCs to the Framingham risk score.

## AUTHOR CONTRIBUTIONS

**Ting‐Yi Tien:** Data curation (equal); formal analysis (equal); methodology (equal); project administration (equal). **Yih‐Jer Wu:** Conceptualization (equal); data curation (equal); formal analysis (equal). **Chiung‐Yin Chang:** Data curation (equal); formal analysis (equal); investigation (equal); methodology (equal). **Chung‐Lieh Hung:** Data curation (equal); project administration (equal). **Yi‐Nan Lee:** Data curation (equal); formal analysis (equal). **Hsin‐I Lee:** Data curation (equal); formal analysis (equal). **Yen‐Hung Chou:** Methodology (equal). **Chao‐Feng Lin:** Data curation (equal); project administration (equal). **Chun‐Wei Lee:** Project administration (equal). **Cheng‐Huang Su:** Conceptualization (lead); data curation (equal); formal analysis (lead); funding acquisition (lead); investigation (lead); methodology (lead); project administration (equal). **Hung‐I Yeh:** Conceptualization (equal); data curation (equal); formal analysis (equal); funding acquisition (equal).

## CONFLICT OF INTEREST STATEMENT

The authors declare no conflict of interests associated with this manuscript.

## Supporting information


Figure S1.

## Data Availability

The data that support the findings of this study will be available from the corresponding author upon reasonable request.
