# Supplementary material for: Hsa‐miR‐134‐5p predicts cardiovascular risk in circulating mononuclear cells and improves angiogenic action of senescent endothelial progenitor cells
Source: J Cell Mol Med. 2024 Jul 3;28(13):e18523. doi: 10.1111/jcmm.18523 (PMC11220343; doi:10.1111/jcmm.18523)
Supplement: Supplementary file 1 — Figure S1. [file JCMM-28-e18523-s001.zip › jcmm18523-sup-0002-Legend.docx]

**Appendices:**

SUPPLEMENTARY FIGURE 1. Analysis of miR-134-5p-overexpressed senescent EPC-secreted cytokines using a human cytokine array and enzyme-linked immunosorbent assay (ELISA) of IL 7. (A) The right panels show data of miR-134-5p-overexpressed senescent EPC-secreted cytokines, and the left panels show miRNA mimic negative control (NC) data (EPCs without miR-134-5p overexpression). Upper two panels are array C6 membranes and lower 2 panels are array C7 membranes. Among the 120 candidate cytokines, EPC-secreted TGF-β1 (C6 membranes), IL-7 (C6 membranes), and TNF-α (C6 membranes) were the three significantly changed by miR-134-5p overexpression, (B) ELISA showed no difference of IL-7 levels respectively in young and senescent EPCs between those with overexpression and those without overexpression (NC group) of miR-134-5 (n = 4). IL-7, interleukin-7. TGF-β1, transforming growth factor beta 1. TNF-α, tumour necrosis factor alfa.
