# Supplementary figures and images for: Hsa‐miR‐134‐5p predicts cardiovascular risk in circulating mononuclear cells and improves angiogenic action of senescent endothelial progenitor cells
Source: J Cell Mol Med. 2024 Jul 3;28(13):e18523. doi: 10.1111/jcmm.18523 (PMC11220343; doi:10.1111/jcmm.18523)

(A)

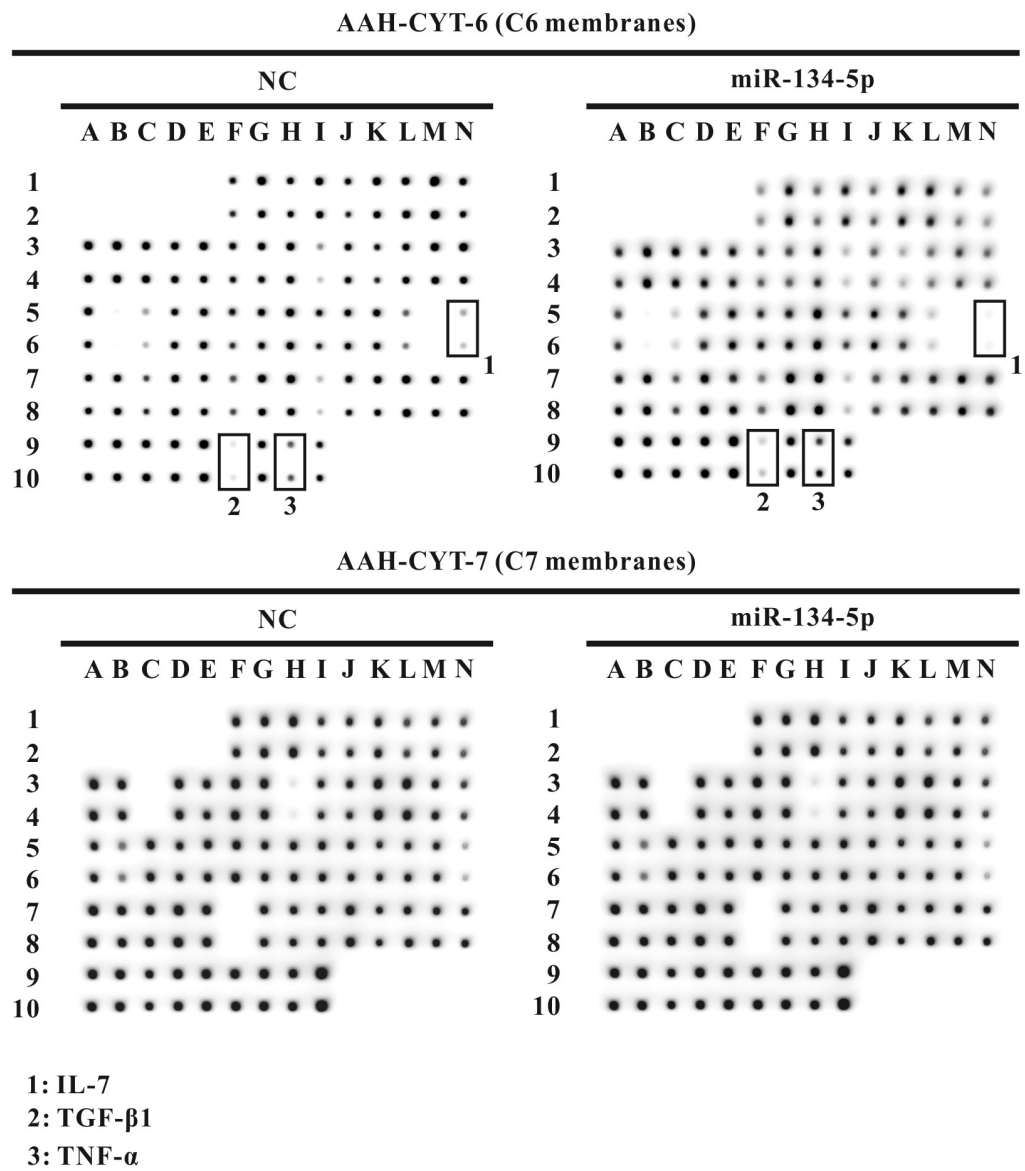

(B)

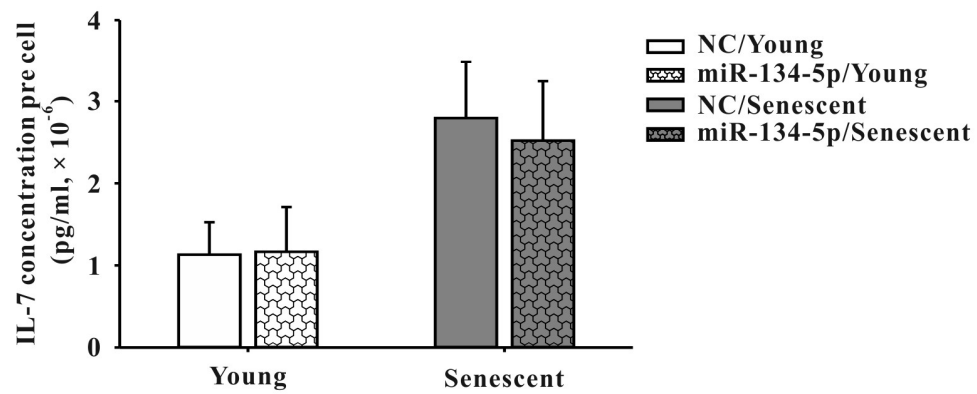

Supplement: Supplementary file 1 — Figure S1. [file JCMM-28-e18523-s001.zip › jcmm18523-sup-0001-AppendixS1.pdf]
